# Enhanced Adenovirus Vaccine Safety Surveillance in Military Setting, United States

**DOI:** 10.3201/eid2906.230331

**Published:** 2023-06

**Authors:** John Iskander, Scott Blanchet, Caitlin Springer, Patrick Rockwell, Dana Thomas, Satish Pillai

**Affiliations:** United States Coast Guard, Washington, DC, USA (J. Iskander, D. Thomas, S. Pillai);; United States Coast Guard Academy, New London, Connecticut, USA (S. Blanchet, C. Springer, P. Rockwell)

**Keywords:** adenovirus, vaccine, vaccination, vaccine safety, safety surveillance, military training, military recruits, adverse effects, enteric infections, respiratory infections, viruses, US Coast Guard, United States

## Abstract

The US Coast Guard Academy began adenovirus vaccination of incoming cadets in 2022. Of 294 vaccine recipients, 15%–20% had mild respiratory or systemic symptoms within 10 days postvaccination but no serious adverse events after 90 days. Our findings support the continued use of adenovirus vaccines in congregate military settings.

Adenovirus infection results in considerable illness among congregate military populations (*1*). US Food and Drug Administration–approved use of a live, oral, bivalent adenovirus vaccine for military populations began in 2011 and was associated with substantial decreases in adenovirus infection incidence at US military basic training centers (*2*,*3*). The US Naval Academy introduced adenovirus vaccination in 2018 after a large adenovirus outbreak (*4*). An adenovirus outbreak involving ≈300 cadets occurred at the US Coast Guard Academy (CGA) in 2019 (*5*). Adenovirus vaccines were introduced for incoming first-year CGA cadets on June 28, 2022. To supplement postlicensure data on adenovirus vaccine safety (*6*), we monitored postvaccination signs and symptoms in those cadets.

We developed a monitoring system to account for military training features, such as restricted cellphone access and a time-sensitive, regimented curriculum. For sick call visits occurring <10 days after vaccination, the CGA clinic used a monitoring tool (Appendix) consisting of 17 postvaccination signs or symptoms obtained from clinical trial results (*7*,*8*). We measured vaccine uptake and inability to swallow pills and monitored cadets for 90 days after vaccination for US Food and Drug Administration–defined serious adverse events (*9*).

Cadets received an in-person briefing from CGA clinic staff on June 27, 2022. The CGA training cadre, with whom the cadets had daily contact, were briefed by clinic leadership on the paper-based reporting tool, reporting requirements, and referring ill cadets to the CGA clinic. Before vaccination, cadets were given the Centers for Disease Control and Prevention adenovirus vaccine information statement and opportunity to ask questions. Subsequently, if cadets sought care for illness at the CGA clinic, staff used the reporting tool to record whether any of the 17 signs and symptoms were present.

During the initial vaccination period (June 28–30, 2022), 293 (97.3%) of 301 first-year cadets received the adenovirus vaccine; 4 (1.3%) cadets were unable to swallow the vaccine. Of 4 cadets isolated for COVID-19 during the initial vaccination period, only 1 subsequently received the vaccine. Of 294 vaccinated cadets, a total of 159 (54.1%) received 1 other vaccine and 53 (18.0%) received >2 additional vaccines.

The average age of the 294 vaccine recipients was 18.25 years; 57% were male, and 43% female. During June 30–July 8, 2022, ≈100 first-year cadets sought care at the CGA clinic for illness, and 65 (22.1%) cadets reported >1 vaccine surveillance sign or symptom. Commonly reported signs and symptoms were cough (20.1%), sore throat (17.0%), headache (16.0%), fatigue (16.0%), nasal congestion (15.3%), and shortness of breath (11.6%) (Table). Frequencies of gastrointestinal symptoms among cadets seeking care at the clinic during the 10-day period after vaccination were 2.3% for abdominal pain, 3.7% for diarrhea, 4.0% for vomiting, and 8.3% for nausea (Table). During the 90 days after vaccination, no serious adverse events were reported, including hospitalization, Guillain-Barre syndrome, or death.

**Table:** Percentages of first-year US Coast Guard cadets reporting signs or symptoms within 10 days after adenovirus vaccination in study of enhanced adenovirus vaccine safety surveillance in military setting, United States*

Signs or symptoms	% Total†
Cough	20.1
Sore throat	17.0
Headache	16.0
Fatigue	16.0
Nasal congestion or rhinorrhea	15.3
Shortness of breath	11.6
Arthralgias or myalgias	10.5
Nausea	8.5
Fever, subjective or measured at >38.0°C	7.8
Increasing weakness in arms or legs	5.8
Numbness or tingling in hands or feet	4.8
Vomiting	4.1
Diarrhea	3.7
Abdominal pain	2.4
New rash	0.7
Dysuria	0.0
Hematuria	0.0

*Total number of vaccinated cadets was 294. †Percentages sum to >100% because vaccine recipients reported multiple signs or symptoms.

During the enhanced postimmunization monitoring period, a concomitant COVID-19 outbreak affected ≈20% of first-year cadets. Of the 65 students who sought care at the CGA clinic and had >1 enhanced surveillance sign or symptom, 8 (12.3%) also tested positive for COVID-19.

The live adenovirus vaccine was well-tolerated; only 4 vaccination failures occurred, the cadets who could not swallow the medication. Of 294 vaccinated CGA cadets, <25% reported signs or symptoms during the monitoring period. Phase 1 adenovirus vaccine trial data (*7*) showed symptom occurrence reached 33% within 8 weeks postvaccination. The most common signs and symptoms reported among CGA first-year cadets corresponded with those noted in a large phase 3 adenovirus trial (*8*), including headache, sore throat, nasal congestion, and cough. In this cohort, rates for gastrointestinal symptoms (2.3%–8.3%) were lower than those reported in the phase 3 trial (*8*).

Shortness of breath in 11.6% of cadets did not clearly correspond to signs and symptoms identified in phase 1 or phase 3 trial safety data. Given that >25% of nonhospitalized adults with COVID-19 report shortness of breath (*10*), the concurrent COVID-19 outbreak might have contributed to reports of this specific symptom.

In conclusion, enhanced passive monitoring established after introducing adenovirus vaccination for incoming first-year CGA cadets did not identify any serious adverse events. Even with the receipt of multiple vaccines and an intercurrent COVID-19 outbreak, the signs and symptoms profile among cadets who had sick calls during the 10-day postvaccination period appears consistent with profiles reported in previous adenovirus vaccine trials. A positive COVID-19 test was observed for 12.3% of cadets who completed the surveillance questionnaire during their sick call, which might explain the 11.6% of cadets who reported shortness of breath. Our favorable real-world findings support continuing adenovirus vaccination of incoming CGA cadet classes and wider use of the vaccine in congregate military settings.

Appendix Additional information for enhanced adenovirus vaccine safety surveillance in military setting, United States.
