# Supplementary material for: Enhanced Adenovirus Vaccine Safety Surveillance in Military Setting, United States
Source: Emerg Infect Dis. 2023 Jun;29(6):1283–5. doi: 10.3201/eid2906.230331 (PMC10202855; doi:10.3201/eid2906.230331)
Supplement: Appendix — Additional information for enhanced adenovirus vaccine safety surveillance in military setting, United States. [file 23-0331-Techapp-s1.pdf]

Article DOI: https://doi.org/10.3201/eid2906.230331

EID cannot ensure accessibility for supplementary materials supplied by authors. Readers who have difficulty accessing supplementary content should contact the authors for assistance.

Enhanced Adenovirus Vaccine Safety Surveillance in Military Setting, United States

Appendix

Adenovirus vaccine postvaccination signs and symptoms reporting tool (day 0 is day of vaccination).

Symptom (Y/N)	Day 0	Day 1	Day 2	Day 3	Day 4	Day 5	Day 6	Day 7	Day 8	Day 9	Day 10
Fever (subjective or measured at >100.5°F)											
Headache											
Fatigue											
Body aches or joint pains											
Cough											
Shortness of breath											
Sore throat											
Nasal congestion or runny nose											
Nausea											
Vomiting											
Diarrhea											
Abdominal pain											
Burning with urination											
Blood in urine											
New rash											
Numbness or tingling in hands or feet											
Inability or increasing weakness in arms or legs											
